# Ultrasound‐Induced Synchronized Neural Activities at 40 Hz and 200 Hz Entrained Corresponded Oscillations and Improve Alzheimer's Disease Memory

**DOI:** 10.1111/cns.70351

**Published:** 2025-04-09

**Authors:** Jiamin Chen, Xingran Wang, Xin Li, Xiaoli Li, Yiyao Zhang, Yi Yuan

**Affiliations:** ^1^ School of Electrical Engineering Yanshan University Qinhuangdao China; ^2^ Key Laboratory of Intelligent Rehabilitation and Neuromodulation of Hebei Province Yanshan University Qinhuangdao China; ^3^ Guangdong Artificial Intelligence and Digital Economy Laboratory (Guangzhou) Guangzhou China; ^4^ School of Automation Science and Engineering South China University of Technology Guangzhou China; ^5^ Neuroscience Institute, NYU Langone Health New York New York USA

**Keywords:** Alzheimer's disease, memory, neural activities, sharp wave ripples, transcranial ultrasound stimulation

## Abstract

**Aims:**

Neurological diseases like Alzheimer's disease (AD) with cognitive deficits show impaired theta, gamma, and ripple bands. Restoring these oscillations may be crucial for rescuing cognitive functions. Low‐intensity transcranial ultrasound stimulation (TUS), a noninvasive neuromodulation method, offers high spatial resolution and deep penetration. However, it remains unclear how 40 Hz and 200 Hz TUS may improve memory in AD by regulating hippocampal oscillations.

**Methods:**

We applied 40 Hz and 200 Hz TUS to the CA1 region of AD mice, performing memory assessments and CA1 electrophysiology recordings simultaneously.

**Results:**

Our results showed that both 40 Hz and 200 Hz TUS significantly improved memory performance in AD mice by targeting the dorsal hippocampus and increasing power in corresponding frequency bands. Specifically, 40 Hz TUS enhanced gamma and ripple bands, while 200 Hz TUS strongly affected both. This enhancement increased during stimulation and persisted 5 days poststimulation. Improved coupling between theta and gamma oscillations indicated better hippocampal coordination with other brain regions. Additionally, 40 Hz TUS raised sharp wave ripple (SPW‐Rs) incidence, and 200 Hz TUS increased both SPW‐R incidence and duration, contributing to memory improvement. Behavioral performance significantly improved with TUS at both frequencies.

**Conclusion:**

Ultrasound‐induced synchronized neural activities at 40 Hz and 200 Hz entrained corresponding oscillations and improved memory in Alzheimer's disease.

## Graphical abstract

Both 40 Hz and 200 Hz TUS enhanced memory and sustained improvements in AD mice. Theta‐gamma PAC, phase‐locked interneuron firing, and SPW‐Rs in the CA1 of AD mice were selectively modulated by 40 Hz and 200 Hz TUS.

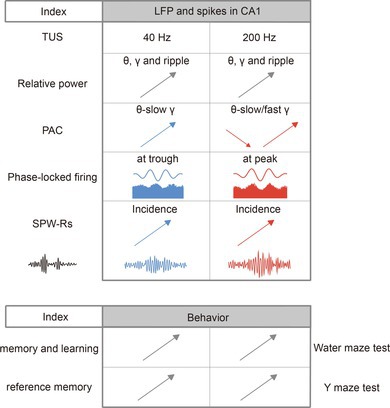

## 1 Introduction

The balanced synchronous activity of neurons is a fundamental mechanism underlying synaptic plasticity, crucial for information processing and consolidation across different brain states [1, 2]. Dysregulation of this synchrony, particularly at the “right” timings, is a hallmark of psychiatric and neurological disorders. Neuronal synchronization manifests as specific frequency bands in local field potentials, known as “oscillations”, such as “Gamma” (30–100 Hz) in the neocortex and hippocampus, or “Ripple” (140–250 Hz) in the hippocampus, often accompanied by high‐frequency events in downstream areas [3, 4]. During active exploration, sensory stimuli transiently synchronize at the “40 Hz” gamma band, facilitating the integration of multimodal information into theta‐nested gamma oscillations in the hippocampus [5]. During rest periods following exploration, hippocampal neurons reactivate within a brief window around the “200 Hz” ripple band, selectively transmitting information to deep regions like the lateral entorhinal cortex or lateral septum [6, 7]. Both gamma and ripple oscillations rely on a delicate balance between excitation and inhibition [8, 9]. This balance renders the hippocampus particularly susceptible to early dysfunction in neurodegenerative processes, notably Alzheimer's disease [10, 11]. Therefore, targeting specific frequency bands in hippocampal neural activity holds promise for modulating cognitive functions in pathological conditions.

In recent years, the idea and accumulating evidence of entraining gamma oscillations at “40 Hz” in the brain have garnered attention as a potential therapy for Alzheimer's disease [10, 12, 13]. However, this has also sparked rising concerns regarding whether the sensory stimulation in the original research effectively involves critical subcortical brain regions, such as the hippocampus‐ the primary relay station for neuronal traffic from diverse brain regions encoding sensory stimuli [14, 15]: light‐flickering stimulation combined with exercise strongly modulates spike activity at 40 Hz in the visual cortex, with less impact observed in the entorhinal cortex and no discernible “gamma” modulation in the hippocampus [16]. On the other hand, widespread auditory [17], tACS [18], or optogenetic stimulation [10] at 40 Hz in targeted areas has shown memory significance in humans and mice, although the alteration of brain oscillations was not evaluated. Thus, a strong, noninvasive approach that enables gamma‐ or ripple‐band oscillation in targeting deep brain areas, such as the hippocampus, to investigate how the neural network is being modulated during stimulation is necessary.

Low‐intensity transcranial ultrasound stimulation (TUS), recently “rediscovered” as a potential brain therapy technique, offers the advantages of noninvasiveness and flexibility in both spatial and temporal dimensions [19, 20]. Consequently, it is emerging as one of the most promising brain stimulation approaches. While chronic TUS pulses at 40 Hz have shown significant reductions in Aβ plaque [21, 22], the underlying cognitive functions and neural mechanisms remain to be carefully investigated. To delve into the effectiveness and underlying mechanisms of ultrasound treatment for Alzheimer's disease, we applied focused stimulation at 40 Hz gamma band and measured neural activities via large‐scale in vivo electrophysiological recording in the hippocampus. Furthermore, we also tested the effect of stimulation at the 200 Hz “Ripple” band in the hippocampus to compare the differences.

## 2 Materials and Methods

### 2.1 Animal and Groups

A total of 48 mice were used, including 12 C57BL/6J and 36 APP/PS1 mice (adult male, 20–25 g; Beijing HFK Bioscience Co. Ltd., China). Mice were housed individually in a well‐ventilated room at 22°C–25°C with a 12‐h light/dark cycle, with free access to food and water. All procedures followed the guidelines of the Animal Ethics and Administrative Council of Yanshan University.

#### 2.1.1 Behavioral Experiment

Six C57BL/6J mice served as controls in the Control group. Eighteen APP/PS1 mice were randomly assigned to the AD (*n* = 6), AD+TUS1 (*n* = 6, 40 Hz ultrasound), and AD+TUS2 (*n* = 6, 200 Hz ultrasound) groups.

#### 2.1.2 Behavioral and Local Field Potential (LFP)/spike Recording Experiment

Six C57BL/6J mice were used as controls in the Control group. Eighteen APP/PS1 mice were divided into AD (*n* = 6), AD+TUS1 (*n* = 6, 40 Hz ultrasound), and AD+TUS2 (*n* = 6, 200 Hz ultrasound) groups. All mice were implanted with 16‐channel nickel‐chromium electrodes in CA1, and behavioral and LFP/spike recordings were conducted simultaneously.

### 2.2 Surgery for Electrode Implantation

Before surgery, mice were anesthetized in a 2% isoflurane induction chamber (0.5 L/min oxygen flow) and transferred to a mouse adapter (68,030, Reward Co., Shenzhen, China), with anesthesia maintained at 1.5% isoflurane. Erythromycin ointment was applied to protect the eyes. A 0.8 mm hole was drilled in the skull above CA1, and a 16‐channel nickel‐chromium microelectrode (AP = −2 mm, ML = 1.35 mm, DV = −1.25 mm; 300–900 kΩ, 4 × 4 array, 35 μm internal diameter, Stablohm 675, CFW, USA) was implanted using a stereotaxic device (ST‐5ND‐C, Stoelting Co., USA). Ground and reference electrodes were attached to screws on the cerebellar skull, and a plastic tube was placed in the ultrasound path. The electrode and tube were secured with dental cement. After surgery, mice were placed on a heating pad until mobile, then returned to their cage for a one‐week recovery before behavioral testing and simultaneous LFP and spike recordings.

### 2.3 Acquisition of LFP and Spikes

A multichannel signal acquisition system (Apollo, Bio‐Signal Technologies, McKinney, TX, USA) was used to record LFP and spikes. The equipment was housed in a copper wire mesh cage to minimize electrical noise interference. Mice underwent simultaneous LFP recordings during the behavioral test. We referred to previous literature [23, 24, 25] and implanted microelectrodes into the CA1 region of the hippocampus. Dental cement was used to secure the electrodes, ensuring stable LFP and spike recordings during the behavioral test. Data were collected from freely moving mice for 1 h before stimulation. Awake mice were then stimulated with TUS for 1 h, followed by continued LFP and spike recordings.

### 2.4 Morris Water Maze Test

The device was a 60 cm diameter, 50 cm height cylinder with quadrant markers, with a smaller 10 cm diameter, 20 cm height cylinder at the center of the target quadrant. Water depth was 21 cm, and temperature was 23°C ± 3°C. Milk was added for contrast against the black mice, and a black cloth bracket minimized external interference. The experiment had two phases: directional navigation and space exploration. Directional navigation lasted 6 days with 4 daily sessions, where mice were randomly placed facing the pool wall and timed to find the platform. Mice remained on the platform for 5 s upon reaching it. Sessions lasted 60 s with rest periods of up to 10 min. Mice that failed to find the platform were guided and stayed for 15 s. Video was captured using a CCD, and data were analyzed following Samson et al.'s method [26], assessing time in quadrants, platform crossings, swimming speed, and trajectory via Matlab.

### 2.5 Y‐Maze Test

The Y‐maze [27] had a white arm measuring 45 cm in length, 10 cm in width, 25 cm in height, and a 120° arm angle. It was placed in a quiet room with constant soft lighting and no external interference. The arms were labeled A, B, and C. The experiment had two parts: spontaneous alternation and new arm exploration [28]. Mice first adapted to the maze for 30 min. The closed arms were designated as new arms, and the mice explored the two open arms for 10 min. Videos of mice exploring all three open arms were collected the next day. After each session, the maze was wiped with 75% alcohol to prevent scent interference.

### 2.6 TUS System and Parameters

In the TUS system, a signal from one function generator, modulated by another (AFG3022C, Tektronix, USA), was amplified by a linear RF power amplifier (E&I240L, ENI Inc., USA) and transmitted to the ultrasound transducer (V302‐SU, Olympus, USA), generating ultrasound waves. CA1 was targeted through a collimator filled with coupling fluid and a plastic tube outside the skull. TUS parameters were: TUS1: FF = 1 MHz, SD = 10 s, PRF = 40 Hz, DC = 5%; TUS2: FF = 1 MHz, SD = 10 s, PRF = 200 Hz, DC = 5%. Total TUS time was 1 h. The sound pressure in both TUS1 and TUS2 was 0.36 MPa, with Isppa at 4.3 W/cm [2] and Ispta at 217.2 mW/cm [2].

### 2.7 Data Quantification

The collected data were divided into four segments: one hour before TUS (Pre) and 2, 8, and 19 h after TUS (2 h, 8 h, 19 h), with each segment lasting 1 h. The sampling rate of the original LFP and spike signals was 30 kHz.

### 2.8 Classification of Awake, NREM and REM State

The preprocessed signal was filtered with a Butterworth bandpass filter (0.1–250 Hz) and analyzed using a 5 s window and 2.5 s step size to obtain delta (0.5–4 Hz), theta (5–10 Hz) [29], and 21–300 Hz segmented power representing electromyographic characteristics. Signals from nonadjacent channels (2, 11, 16) were selected, and FIR Butterworth bandpass filtering (275, 300, 975, 1000) removed low‐frequency LFPs. The normalized linear correlation between two channels represented electromyography. Sleep states were classified as follows: [30] (1) NREM: delta power above the threshold, electromyographic power below the mean plus one standard deviation; (2) REM: delta power below the threshold, theta/delta deviating by more than one standard deviation, electromyographic power below the mean plus one standard deviation; (3) Awake: all other states. Mouse activity videos were used to confirm state assignment.

### 2.9 Analysis of Power and PAC of LFP

The power of the LFP was calculated using the Welch function with a 2 k window and nonoverlapping segments. The total power range for relative power was 0.5–200 Hz. PAC was calculated using the phase‐locking value algorithm [31]. The modulation index (MI) of PAC was expressed by the following equation:

(1) $$\mathrm{MI}=\left|\frac{1}{N}\sum_{t=1}^{N}e^{i\left(\Phi_{\text{lowfreq}}\left(t\right)-\Phi_{\text{highamp}}\left(t\right)\right)}\right|$$

where *N* represents the size of the signal, $\Phi_{\text{lowfreq}}\left(t\right)$ represents the phase of the low‐frequency signal, and $\Phi_{\text{highamp}}\left(t\right)$ represents the phase of the amplitude of the high‐frequency signal modulated by the low‐frequency signal. We calculated the MI of theta‐slow gamma and theta‐fast gamma PAC.

### 2.10 Calculation of SPW‐Rs

Sharp wave ripples (SPW‐Rs) were extracted from the LFP during NREM sleep based on previous literature [23]. The LFP was first band‐pass filtered between 130 and 200 Hz. The segmented root mean square was then computed using a 300‐length window and a 30‐length step size. A moving average filter with a window length of 11 was applied to calculate the mean. Signals exceeding the mean by 2 standard deviations were considered the start and end points of candidate ripple events. Incomplete waveforms were discarded, and adjacent events with intervals under 30 ms were merged. The incidence and duration of SPW‐Rs were calculated.

### 2.11 Analysis of Spikes

Electrophysiological signals were high‐pass filtered (> 300 Hz) with an 80 μV threshold. Spike emission types were classified using waveform and autocorrelation diagrams in BOSS. Phase locking was calculated based on spike correspondence to theta and gamma waveforms.

### 2.12 Statistical Analysis

Statistical analysis was conducted using MATLAB (The MathWorks Inc., MA, USA). Analysis of variance (ANOVA) or Kruskal–Wallis was used to compare data across groups and control for Type I error inflation. Tukey's Honest Significant Difference (HSD) post hoc test was performed to identify significant differences between groups.

## 3 Results

### 3.1 Characterization of TUS and Neural Activity From CA1

The TUS system used in this study is the same as in our previous work [32] (Figure S1A). Given CA1's key role in learning and memory, it was chosen as the stimulation target and LFP collection site [33]. The ultrasound transducer was positioned based on the sound field distribution to focus the beam on the target (Figure S1C). Two pulse repetition frequencies, corresponding to gamma (TUS1) and ripple (TUS2) oscillations, were applied for 1 h daily over 6 days to investigate TUS effects on neural activity and memory in AD mice (Figure S1B). Theta, gamma, SPW‐Rs, and neuronal action potentials from CA1 were recorded (Figure S1E,F).

### 3.2 TUS Improves Memory Abilities of AD Mice

**FIGURE 1:**
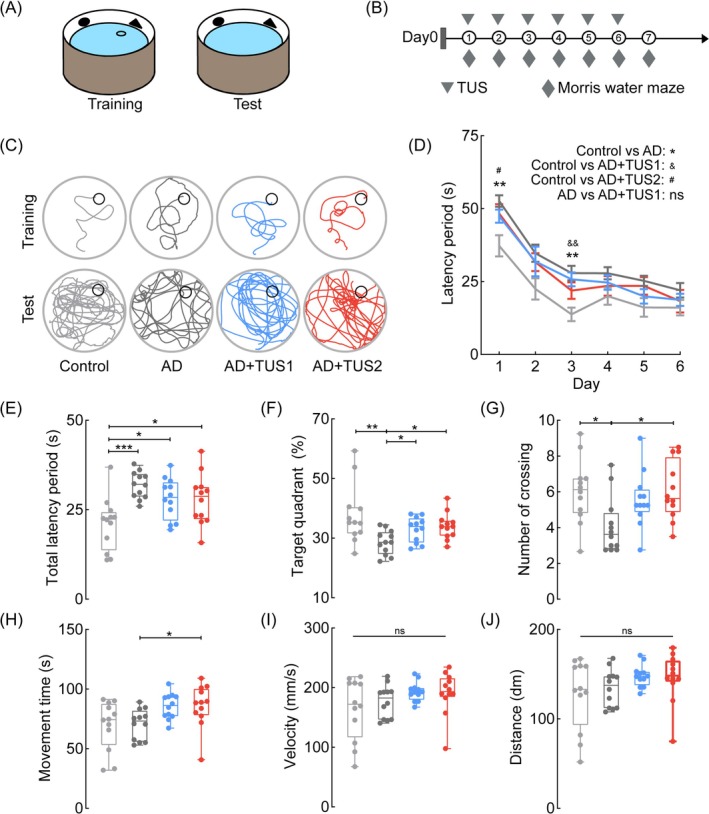
TUS improves memory abilities of AD mice. (A) Schematic diagram of the Morris water maze device. (B) Timing diagram of TUS and Morris water maze test. (C) The movement trajectories of different groups on the search platform (blue line: TUS1 group (40 Hz TUS), red line: TUS2 group (200 Hz TUS)). The control group exhibited a more “linear” search pattern, with the highest frequency of exploration in the target quadrant and the greatest number of passes over the platform once removed. In contrast, the AD group predominantly displayed “peripheral” search patterns, spending the most time searching for the platform. (D) Latency periods of each day for each group (**p < 0.01，&&*p* < 0.01，#*p* < 0.05，ns: *p* > 0.05). The AD group took the longest time to find the platform, while the control group took the least time, with the TUS group falling in between. (E) Total latency period statistics for each group. The overall average latency over the 6‐day period followed a similar trend to that observed on individual days. (F) Percentage of time spent in the target quadrant of each group. The control group spent the most time in the target quadrant, followed by the TUS group, and the AD group spent the least. (G) The number of platform crossing of each group. The control group crossed the target area most frequently, with the TUS group second, and the AD group the least. (H–J) Movement time, movement speed and distance traveled of mice from each group. No differences in motor ability were observed among the groups. ANOVA or the Kruskal–Wallis test, followed by Tukey–Kramer post hoc multiple comparisons. *n* = 12 mice in each group, *p < 0.05, **p < 0.01, ***p < 0.001.

To assess whether 40 Hz and 200 Hz TUS improve memory in AD mice, we used the Morris water maze (Figure 1A,B). AD mice showed significantly increased escape latency and route length during the learning phase compared to wild‐type controls (Figure 1C). Our findings suggest that 40 Hz and 200 Hz TUS enhance learning performance in the Morris water maze, as indicated by reduced escape latency (Figure 1D,E; ANOVA, *n* = 12, *p < 0.05, **p < 0.01, ***p < 0.001). On day 7, memory retention tests revealed no group differences in locomotor ability (Figure 1H–J). AD+TUS1 (40 Hz) and AD+TUS2 (200 Hz) mice spent more time in the target quadrant and crossed the platform more frequently than untreated AD mice (Figure 1F,G; ANOVA, *n* = 12, *p < 0.05, **p < 0.01). No differences were observed between the TUS1 and TUS2 groups, suggesting both 40 Hz and 200 Hz TUS enhance memory formation. Although current data support a positive effect of TUS on learning, the underlying mechanisms of memory enhancement require further investigation through electrophysiological studies.

### 3.3 TUS Maintains Memory Improvements in AD Mice

**FIGURE 2:**
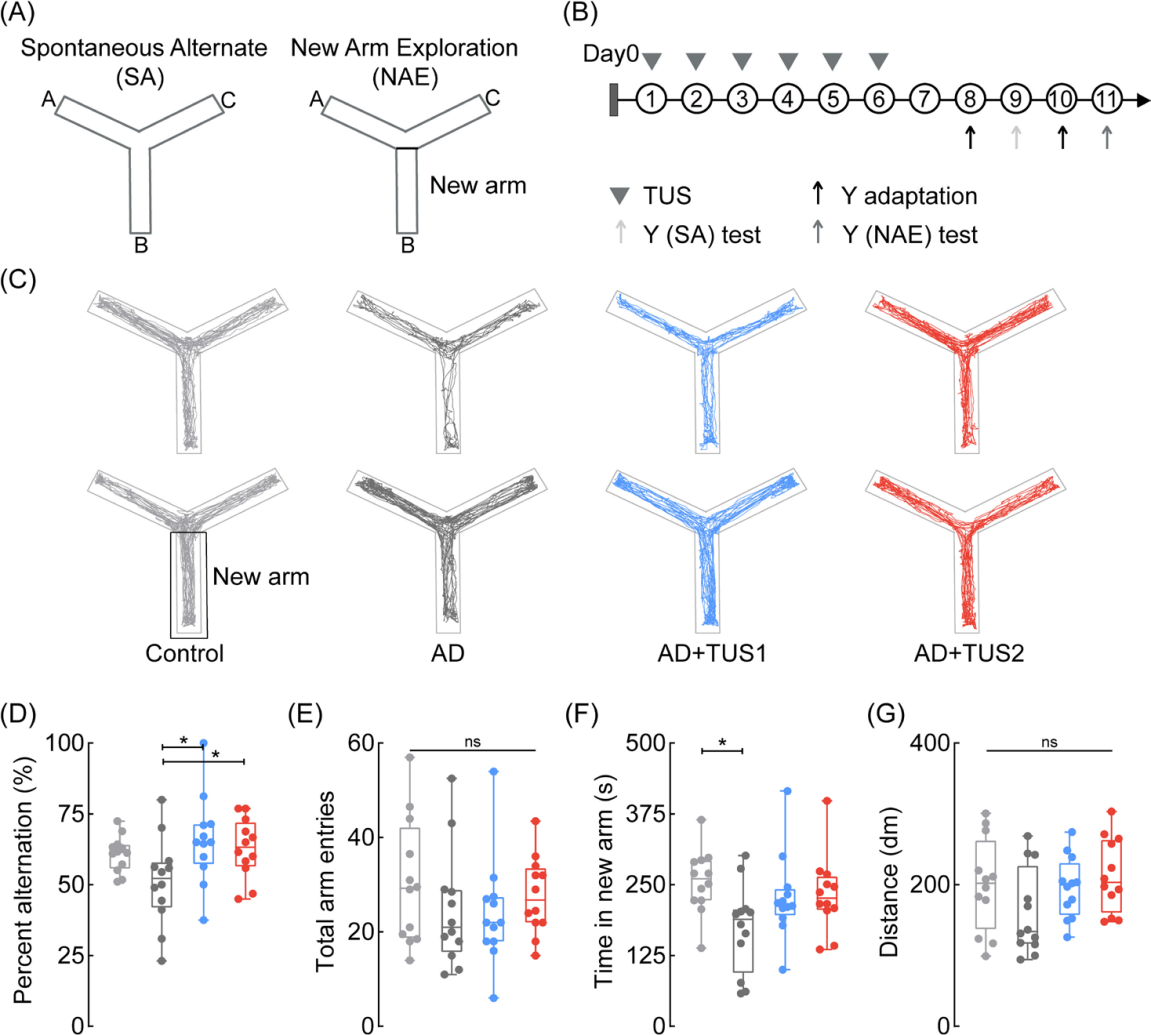
TUS maintains memory improvements in AD mice. (A) Schematic diagram of the Y‐maze test. (B) Timing diagram of TUS and the Y‐maze test. (C) Spontaneous movement trajectories of mice in each group. Top: The spontaneous alternation experiment. In the control group, mice explored the three arms more evenly, whereas the AD group tended to neglect one arm during exploration. Bottom: The new arm exploration experiment. The “novel arm” refers to the arm that was not explored during the habituation phase. Mice in the control group spent more time exploring the novel arm, while those in the AD group preferred a more free‐form exploration approach. (D, E) Spontaneous alternation rate and number of entries and exits for each group. The spontaneous alternation rate, which reflects the extent of balanced exploration across the arms, was higher in the control group than in the AD group. The total number of arm entries indicated the overall exploratory activity, with no significant difference in exploratory behavior between the groups. (F, G) Duration and travel distance in the new arm for each group. Control mice spent more time in the novel arm compared to AD mice. No differences in motor ability were observed between the groups. ANOVA or the Kruskal–Wallis test, followed by Tukey–Kramer post hoc multiple comparisons. *n* = 12 mice in each group, *p < 0.05.

We investigated whether the memory improvement induced by 40 Hz and 200 Hz TUS in AD mice is sustained. A Y‐maze spontaneous alternation test was conducted on days 8–11 after 6 days of TUS (Figure 2A,B). AD mice showed reduced preference for unvisited arms and uneven exploration, indicating impaired memory. After 40 Hz and 200 Hz TUS, AD mice exhibited more balanced arm exploration and significantly increased spontaneous alternation rates (Figure 2C–E; ANOVA, *n* = 12, *p < 0.05). The effects observed in the Y‐maze suggest that TUS treatment may have a lasting impact on memory performance. A new arm exploration test on days 10–11 confirmed this, with AD mice showing fewer visits to the unexplored arm compared to controls (Figure 2F; *p < 0.05). The AD+TUS1 and AD+TUS2 groups showed more frequent and longer explorations than untreated AD mice (Figure 2F,G), with no significant difference between TUS groups. These findings demonstrate that the improvement in memory ability of mice 6 days after TUS can last for 5 days. CA1 neural oscillations are crucial for memory [34]. However, further research, particularly electrophysiological analyses, is needed to assess the impact of TUS on memory enhancement.

### 3.4 TUS Modulates Power of LFP of CA1 Neural Oscillation

**FIGURE 3:**
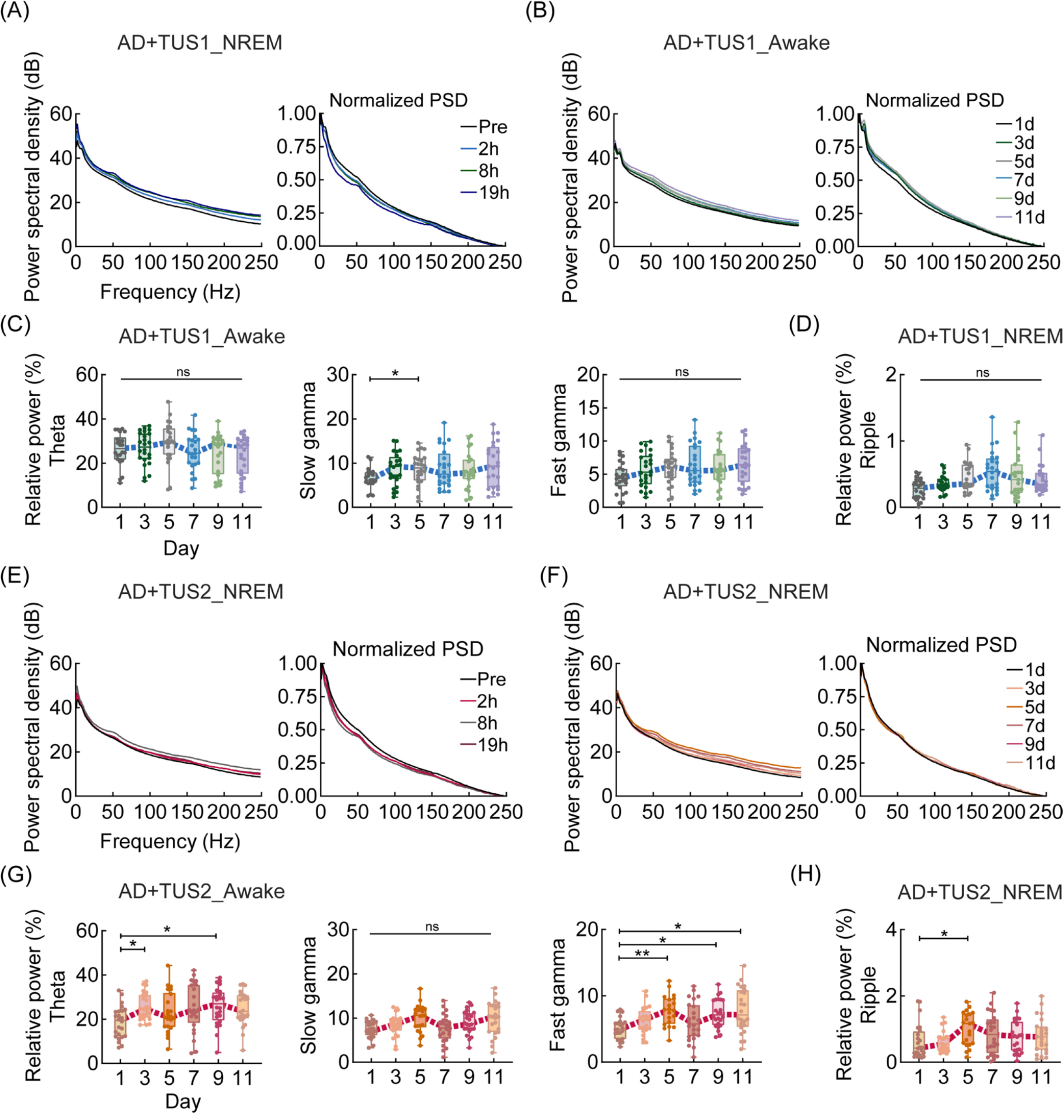
TUS modulates the power of LFP of CA1 neural oscillation. (A) Power spectrum curve of LFP in the NREM state in the TUS1 group (Right: Normalized PSD curve). (B) Power spectrum curve of LFP in the Awake state in the TUS1 group. (C) Relative power of theta (5–10 Hz), slow gamma (30–50 Hz), and fast gamma (50–100 Hz) in the Awake state of the TUS1 group. (D) Relative power of ripple (140–200 Hz) for NREM states in the TUS1 group. (E) Power spectrum curve of LFP in the NREM state in the TUS2 group (Right: Normalized PSD curve). (F) Power spectrum curves of LFP in the NREM state of the TUS2 group. (G) Relative power of theta, slow gamma, and fast gamma oscillations in the Awake state in the TUS2 group. (H) Relative power of ripple oscillation in the NREM states of the TUS2 group. ANOVA or the Kruskal–Wallis test, followed by Tukey–Kramer post hoc multiple comparisons. *n* = 6 mice in each group, *p < 0.05, **p < 0.01.

To investigate these questions, we recorded LFP and spike signals from CA1, analyzing oscillation patterns during awake, NREM, and REM states (Figure S2). Our results show that 40 Hz and 200 Hz TUS generally increased LFP power spectral density in CA1 (Figure 3A,B,E,F; Figure S3, Figure S4). During wakefulness, neither TUS frequency affected relative power across frequency bands at Pre, 2 h, 8 h, or 19 h post‐TUS, but by day 5, theta, slow gamma, and fast gamma power significantly increased (Figure 3C,G; ANOVA, *n* = 6, *p < 0.05, **p < 0.01). In NREM, while power remained unchanged immediately post‐TUS, ripple band power peaked on day 7 and declined by day 11 (Figure 3D,H; ANOVA, *n* = 6, *p < 0.05). No changes were observed during REM. Prior studies suggest impaired theta, slow gamma, and fast gamma power in APP/PS1 mice during wakefulness [35]. Our findings suggest that 40 Hz and 200 Hz TUS modulate the power of LFP of CA1 neural oscillation.

### 3.5 TUS Modulates Theta‐Gamma PAC in CA1

**FIGURE 4:**
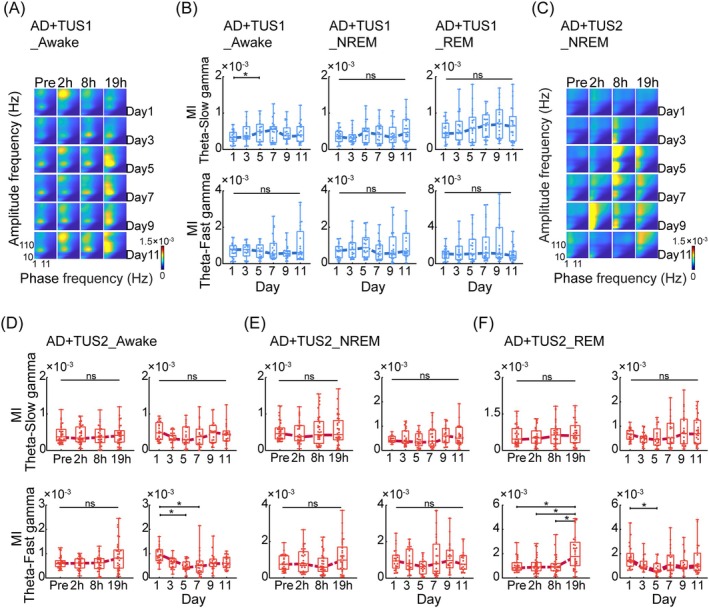
TUS modulates theta‐gamma PAC in CA1. (A) Images of the MI of PAC of the analytical phase (1–20 Hz) and analytical amplitude (10–200 Hz) in the awake state of the TUS1 group. (B) MI of PAC of theta‐slow gamma and theta‐fast gamma over days in awake, NREM, and REM states. Theta‐slow gamma gradually increased over time. (C) Images of the MI of PAC of the analytical phase (1–20 Hz) and analytical amplitude (10–200 Hz) in the NREM state of the TUS2 group. (D–F) The MI of PAC of theta‐slow gamma and theta‐fast gamma in awake, NREM, and REM states (left: Different time periods; right: Different days). Theta‐fast gamma slowly decreased following TUS stimulation. ANOVA or the Kruskal–Wallis test, followed by Tukey–Kramer post hoc multiple comparisons. *n* = 6 mice in each group, *p < 0.05.

Neural oscillations facilitate information transmission and integration. Beyond LFP power, the coordination of oscillations across frequency bands is essential for memory formation and persistence. Specifically, PAC between theta and gamma oscillations in the hippocampus is linked to memory [36]. Impaired theta‐gamma PAC in CA1 is a hallmark of AD, and AD mice exhibited significantly lower theta‐gamma PAC MI compared to controls (Figure S5A,B), consistent with previous findings [37, 38]. We analyzed theta‐gamma PAC across awake, NREM, and REM states. While 40 Hz TUS did not affect theta‐slow or theta‐fast gamma PAC MI at Pre, 2 h, 8 h, or 19 h post‐TUS (Figure S6), 200 Hz TUS increased theta‐fast gamma PAC MI, peaking at 19 h. Over 11 days, theta‐slow and theta‐fast gamma PAC MI initially declined until day 5, then increased with 200 Hz TUS (Figure 4D–F; ANOVA, *n* = 6, *p < 0.05). In contrast, 40 Hz TUS increased theta‐slow gamma PAC MI, peaking on day 7 before gradually diminishing (Figure 4B; *p < 0.05). These findings suggest theta‐slow gamma PAC is frequency‐selective, with 40 Hz TUS enhancing PAC across awake, NREM, and REM states, supporting improved memory in the Morris water maze.

### 3.6 TUS Induces Specific Phase‐Locked Firing to Theta and Gamma Oscillations in CA1

**FIGURE 5:**
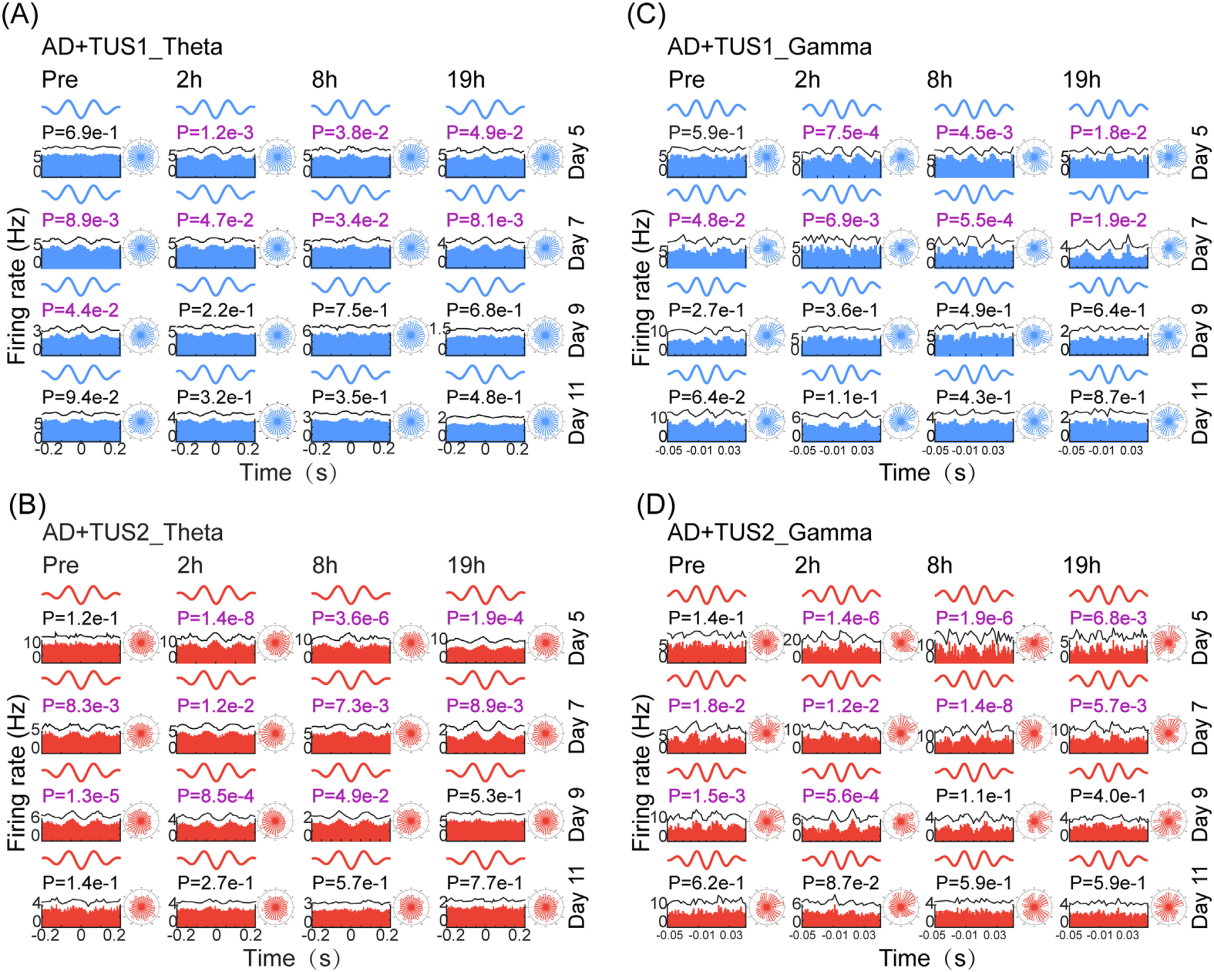
TUS induces specific phase‐locked firing to theta and gamma oscillations in CA1. (A) Phase‐locked firing of interneurons in the trough of theta oscillations in the TUS1 group (Rayleigh's test, *n* = 6 mice). (B) Phase‐locked firing of interneurons in the peak of theta oscillations in the TUS2 group (Rayleigh's test, *n* = 6 mice). (C) Phase‐locked firing of interneurons in the trough of gamma oscillations in the TUS1 group (Rayleigh's test, *n* = 6 mice). (D) Phase‐locked firing of interneurons in the peak of gamma oscillations in the TUS2 group (Rayleigh's test, *n* = 6 mice, *p < 0.05, **p < 0.01, ***p < 0.001, ****p < 0.0001).

Phase‐locked firing of neurons to oscillations in different frequency bands also impacts memory formation [39]. We analyzed the phase‐locked firing of pyramidal neurons and interneurons to theta and gamma oscillations and examined how phase locking of population neurons varies with oscillation peaks and troughs. Under both 40 Hz and 200 Hz TUS, interneurons predominantly fired at the theta trough by day 3 with 40 Hz and day 5 with 200 Hz TUS (Figure 5B, Figure S7A; Rayleigh's test, **p < 0.01, ****p < 0.0001). By day 5, interneurons also fired at the gamma trough under both TUS frequencies (Figure 5C,D; ***p < 0.001, ****p < 0.0001). Under 200 Hz TUS, the firing shifted from the trough to the peak after day 5, persisting until day 11, a shift not seen with 40 Hz TUS. Phase‐locking of pyramidal neurons to theta and gamma oscillations was absent at both frequencies. Interneurons did not exhibit phase‐locking to ripple oscillations (Figure S8). Simultaneously, the firing rates of the population increased during the stimulation period. Quantitative analysis of phase relationships (Figure S9) showed that TUS1 delayed the peak firing of the neuronal population relative to theta/gamma oscillations, whereas TUS2 advanced it. Thus, interneurons' phase‐locked firing to theta and gamma is frequency‐selective, with trough firing under both TUS frequencies potentially enhancing memory in AD mice. The 200 Hz TUS‐induced shift may explain why the memory improvement observed in mice after 6 days of TUS persists for up to 5 days.

### 3.7 TUS Modulates SPW‐Rs in CA1

**FIGURE 6:**
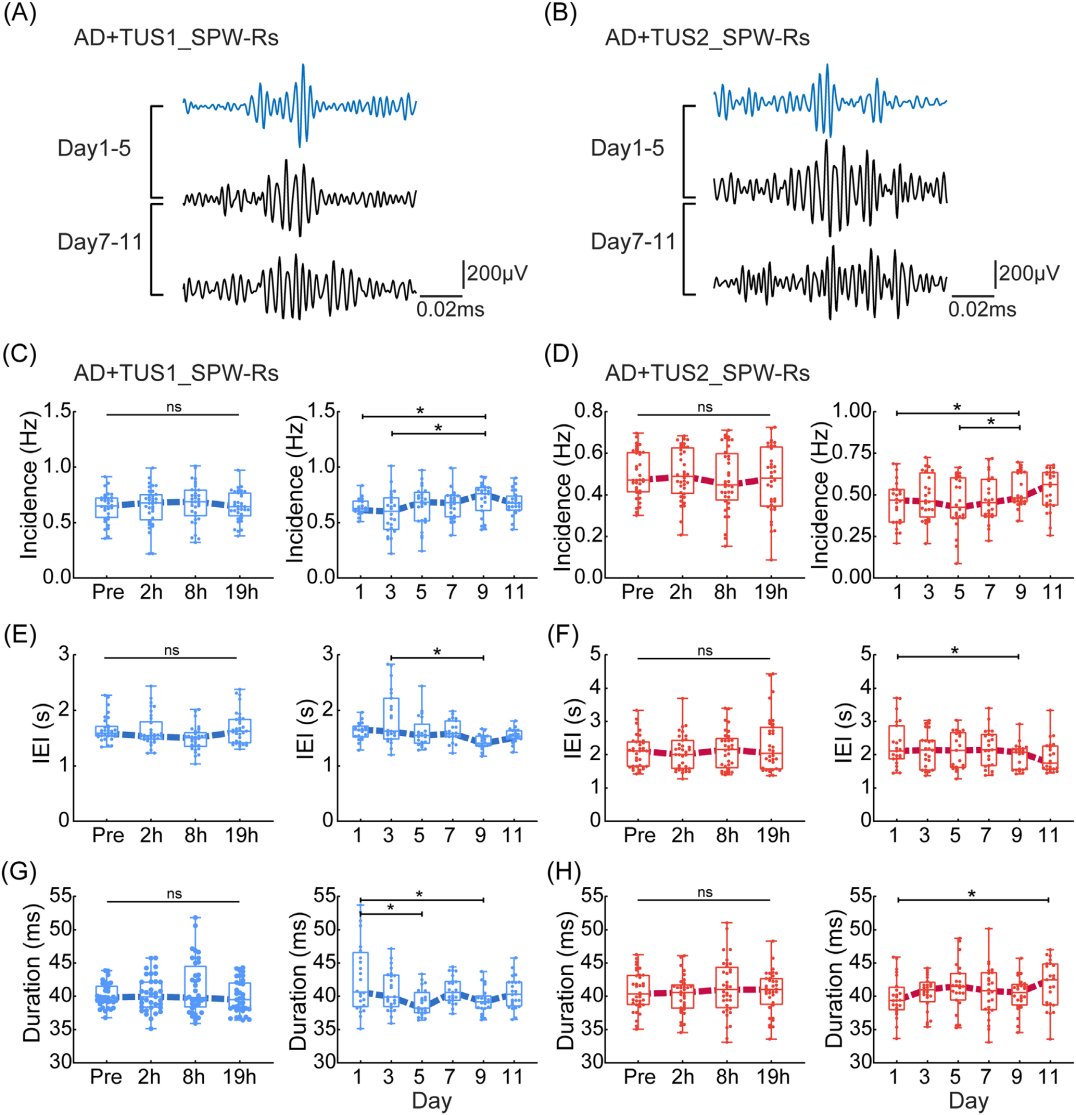
TUS modulates SPW‐Rs in CA1. (A, B) Schematic representation of the changes in the SPW‐Rs over different days. (C, D) Incidence of SPW‐Rs in the TUS1 and TUS2 groups (C: TUS1 group, D: TUS2 group. Left: Different time periods. Right: Different days). The incidence of SPW‐Rs gradually increased within 5 days after the cessation of TUS stimulation. (E, F) Inter‐event interval (IEI) of SPW‐Rs in the TUS1 and TUS2 groups (E: TUS1 group, F: TUS2 group. Left: Different time periods. Right: Different days). (G, H) Duration of SPW‐Rs in the TUS1 and TUS2 groups (G: TUS1 group, H: TUS2 group. Left: Different time periods. Right: Different days). The duration of SPW‐Rs slowly decreased following TUS1 stimulation, but slowly increased following TUS2 stimulation. ANOVA or the Kruskal–Wallis test, followed by Tukey–Kramer post hoc multiple comparisons. *n* = 6 mice in each group, *p < 0.05.

We analyzed SPW‐Rs during NREM, as their incidence and duration in CA1 are crucial for spatial memory consolidation [23, 40]. No significant changes were observed at Pre, 2 h, 8 h, or 19 h post‐TUS under both 40 Hz and 200 Hz conditions (Figure 6C,D,G,H). However, from day 7 to 11, SPW‐Rs incidence and duration significantly increased under 40 Hz TUS (Figure 6C,G; ANOVA, *n* = 6, *p < 0.05), supporting enhanced memory retention. Under 200 Hz TUS, SPW‐Rs duration peaked on day 5 and extended from days 7 to 11 (Figure 6D,H), likely contributing to memory improvement. These findings suggest that 40 Hz TUS increases SPW‐Rs incidence, while 200 Hz TUS prolongs duration, both contributing to memory enhancement in AD mice.

## 4 Discussion

This study applied 40 Hz and 200 Hz TUS to stimulate the CA1 of AD mice, with key findings summarized in Table 1. Both frequencies improved and sustained memory in AD mice, but had different effects on CA1 neural oscillations. Specifically, 40 Hz TUS enhanced theta‐slow gamma PAC, induced and maintained phase‐locked interneuron firing at the trough of theta and gamma waves, and increased SPW‐Rs incidence. In contrast, 200 Hz TUS modulated theta‐slow/fast gamma PAC, maintained interneuron firing at the peak of theta and gamma waves, and increased both SPW‐Rs incidence and duration. These results lay the foundation for exploring 40 Hz and 200 Hz TUS as potential therapeutic strategies for AD.

**TABLE 1:** Main results of 40 Hz and 200 Hz TUS for AD mice.

Experiment	Index	40 Hz TUS	200 Hz TUS
Behavior	Water maze test	Enhanced spatial memory and learning abilities in AD mice	Enhanced spatial memory and learning capacity in AD mice
	Y maze test	Enhanced spatial work and reference memory in AD mice	Enhanced spatial work and reference memory in AD mice
LFP and spikes	Relative power	Increase relative power of theta, gamma and ripple in CA1	Increase the relative power of theta, gamma and ripple in CA1
	PAC	**Theta‐slow gamma PAC increase**	**Theta‐Slow/fast gamma PAC first decreases and then increases**
	Phase‐locked firing	**Phase‐locked firing remains at trough of theta and gamma**	**Phase‐locked firing remains at peak of theta and gamma**
	SPW‐Rs	**Incidence of SPW‐Rs waves increase**	**Incidence and duration of SPW‐Rs waves increase**

*Note:* Significance of bold text highlights a difference between the two sets of parameters.

Both theta and gamma oscillations in the hippocampus are critical for working memory [41], with gamma oscillations playing a role in the further processing of working memory [3], while SPW‐Rs support memory consolidation [42]. We found that both 40 Hz and 200 Hz TUS enhanced the relative power of theta, gamma, and ripple oscillations in CA1, indicating increased activity in these frequency bands. This enhancement likely contributes to the observed improvement in memory abilities in AD mice, suggesting that TUS may positively influence neural oscillations related to memory processing. In WT mice, 40 Hz TUS decreased relative gamma power, whereas 200 Hz TUS had no effect (Figure S11).

We observed distinct patterns of theta‐gamma PAC, interneuron phase‐locking to theta/gamma oscillations, and SPW‐R discharge under 40 Hz and 200 Hz TUS. This aligns with previous findings showing that excitatory and inhibitory neurons respond differently to ultrasound pulse repetition frequency [17]. Excitatory neurons are closely linked to theta oscillations [43], while inhibition is essential for gamma synchronization [44]. SPW‐Rs reflect coordinated neuron firing [45]. Thus, we speculate that the differential sensitivity of neurons to TUS frequencies at 40 Hz and 200 Hz may account for the varied discharge patterns of theta, gamma, and ripple in CA1.

A key question is why 40 Hz and 200 Hz TUS generate distinct theta, gamma, and ripple oscillation patterns, yet both enhance memory and sustain improvement in AD mice. We know that slow gamma oscillations are involved in memory retrieval, activating episodic memory and promoting its preservation by recruiting memory storage units [46]. Initial memory formation relies on the spatiotemporal coordination of theta and gamma oscillations, activating neuronal pathways [47]. The strength of theta‐gamma PAC is positively correlated with working memory enhancement [34, 36]. Thus, the increase in theta‐low gamma PAC may explain why 40 Hz TUS improves memory in AD mice, whereas the delayed effect under 200 Hz TUS suggests a secondary role in memory improvement.

Neuron phase‐locked firing to LFP is crucial for retaining working memory [48]. Increased spike‐LFP phase synchronization is observed during memory processes, such as working memory maintenance and long‐term memory encoding and retrieval. This synchronization serves as a common neural feature in memory functions. For instance, in rodents, sequential item encoding in short‐term memory involves spike phase‐locking to theta oscillations [49]. In our study, both 40 Hz and 200 Hz TUS phase‐locked interneuron firing with theta and gamma oscillations, which may explain their role in enhancing memory in AD mice.

Hippocampal SPW‐Rs play a key role in memory consolidation, as confirmed by previous studies [36]. An increase in SPW‐Rs enhances memory, while prolonged SPW‐Rs duration aids the propagation of SPW‐Rs from CA1 to the cortex, supporting long‐term memory formation and sustaining memory improvement [23, 45, 50]. For instance, closed‐loop optogenetic stimulation has been shown to extend SPW‐Rs, improving memory during maze learning [23]. In our study, 40 Hz TUS increased SPW‐Rs frequency, likely enhancing memory by transferring more information. Additionally, 200 Hz TUS not only increased SPW‐Rs frequency but also prolonged their duration, promoting long‐term memory and sustaining memory improvement.

What is the molecular mechanism by which TUS improves memory in AD mice? In previous studies, researchers have conducted a series of experiments to investigate the effects of TUS on the molecular level in the brain tissue of AD mice and rats. One study showed that TUS alleviates AD pathology and improves cognitive and memory functions [51], significantly reducing aluminum concentration, acetylcholinesterase activity, antibody deposition, and nuclear pyknosis in rat brain tissue caused by AlCl_3_ [52]. In addition, researchers found that TUS slows the shortening of telomeres in the cortex and myocardial tissue of AD mice, thereby improving spatial learning and memory [53]. More importantly, other studies have demonstrated that TUS activates more microglia that co‐localize with amyloid‐beta (Aβ) plaques, significantly reduces Aβ protein content in brain tissue, and improves brain functional connectivity [11, 21, 22]. These studies collectively discuss the molecular effects of TUS on memory in AD mice. In our study, we focused on the modulation of neural oscillations in the CA1 region of AD mice and examined the therapeutic effects of ultrasound from the perspective of neural oscillations. Unlike previous studies, our approach provides a different perspective on the therapeutic effects of ultrasound in AD mice. However, a limitation of our study is that it is more macroscopic in nature, and therefore lacks detailed exploration of the molecular mechanisms. For example, we do not yet know whether or how TUS alters neural oscillations through molecular changes in brain tissue. In future research, we will address these questions in greater depth.

Previous studies suggest that ultrasound, as a mechanical wave, activates mechanosensitive ion channels, including Piezo1 and Piezo2, enhancing neural excitability [54]. Potassium, sodium, and calcium voltage‐gated channels have also been identified as targets [55, 56, 57], contributing to ultrasound‐induced neuronal action potentials. These excitability changes likely alter LFP coding and spike‐LFP coupling, establishing new neural oscillation patterns. Future research will use optogenetic or chemogenetic approaches to verify the ion channel mechanisms involved in TUS regulation of CA1 oscillations.

Currently, noninvasive physical therapy techniques for AD in clinical practice include transcranial magnetic stimulation (TMS) and transcranial direct current stimulation (tDCS) [58, 59]. Research has shown that TMS can affect cellular redox status and the amyloid production process, increase the excitability of the cerebral cortex, promote synaptic plasticity, and enhance the cognitive abilities of AD patients [60]. Multisite TMS and long‐term treatments are also more effective at improving AD‐related cognitive functions [61]. Additionally, previous research has shown that tDCS can significantly modulate cortical electroencephalogram (EEG) activity in AD patients, thereby improving their memory performance and general cognitive function [62]. However, due to the limited penetration depth of magnetic and electric fields, TMS and tDCS cannot effectively stimulate deep brain regions, such as the hippocampus, and are incompatible with MRI for precise navigation [63]. In comparison, TUS offers higher spatial resolution and stimulation depth and can precisely target different deep brain regions. We believe that TUS has the potential to become a new physical modulation tool for the clinical treatment of AD [64, 65].

Previous studies suggest ultrasound stimulation may modulate neural activity via nonspecific auditory responses [66, 67]. In our study, six AD mice were stimulated using specific ultrasound waveforms from prior literature [68]. Results in Figure S10, showing similar effects for rectangular and trapezoidal pulses, indicate that the memory enhancement observed with 40 Hz and 200 Hz TUS in AD mice is not due to auditory influences.

## 5 Conclusion

In conclusion, ultrasound‐induced synchronized neural activities at 40 Hz and 200 Hz entrain corresponding oscillations and improve AD memory.

## Author Contributions

Jiamin Chen: methodology, software, data curation, writing – review and editing, Xingran Wang: methodology, software, data curation, writing – review and editing, Xin Li: methodology, writing – original draft. Xiaoli Li: software, writing – original draft. Yiyao Zhang: conceptualization, data curation, writing – original draft. Yi Yuan: supervision, conceptualization, data curation, writing – review and editing.

## Conflicts of Interest

The authors declare no conflicts of interest.

## Supporting information


Data S1.

## Data Availability

The data and code that underpin the findings of this study are available from the corresponding authors, upon reasonable request.
