# Supplementary material for: Ultrasound‐Induced Synchronized Neural Activities at 40 Hz and 200 Hz Entrained Corresponded Oscillations and Improve Alzheimer's Disease Memory
Source: CNS Neurosci Ther. 2025 Apr 9;31(4):e70351. doi: 10.1111/cns.70351 (PMC11979792; doi:10.1111/cns.70351)
Supplement: Supplementary file 1 — Data S1. [file CNS-31-e70351-s001.zip › Supplementary materials.docx]

**SUPPLEMENTARY MATERIALS**

**
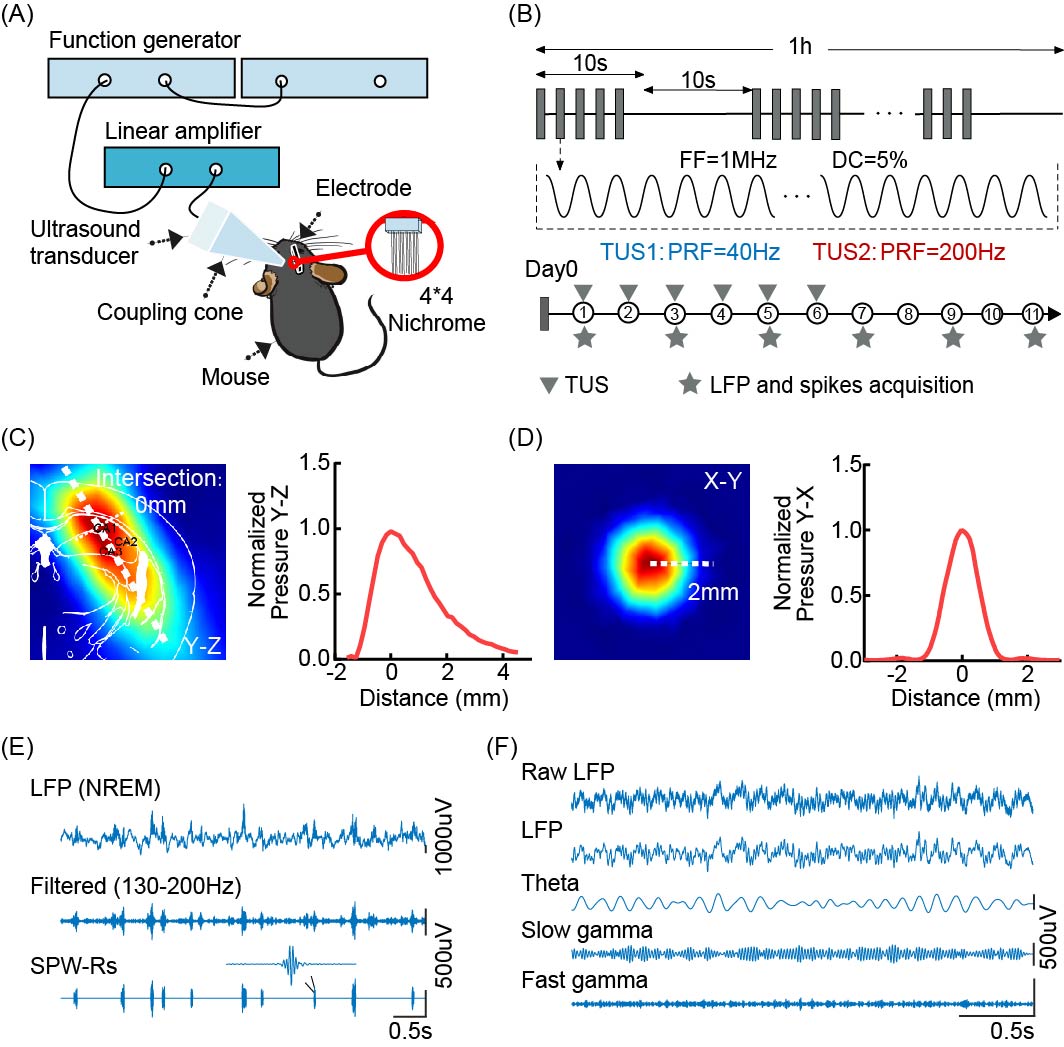
**

**Supplementary Figure 1.** Characterization of TUS and neural activity in CA1. (A) Schematic diagram of the TUS system. (B) Schematic diagram of ultrasound parameters and the timing diagram of TUS. (C) Ultrasound field distribution in the longitudinal section and the sound pressure distribution curve along the direction of ultrasound propagation. The dashed line intersection is the point where the maximum ultrasound pressure can act on CA1. (D) Ultrasound field distribution in the transverse section and sound pressure distribution along the white dotted line. (E) LFP and SPW-Rs in the NREM state from CA1. (F) Theta (5-10 Hz), slow gamma (30-50 Hz), and fast gamma (50-100 Hz) oscillations in the awake state from CA1.

**
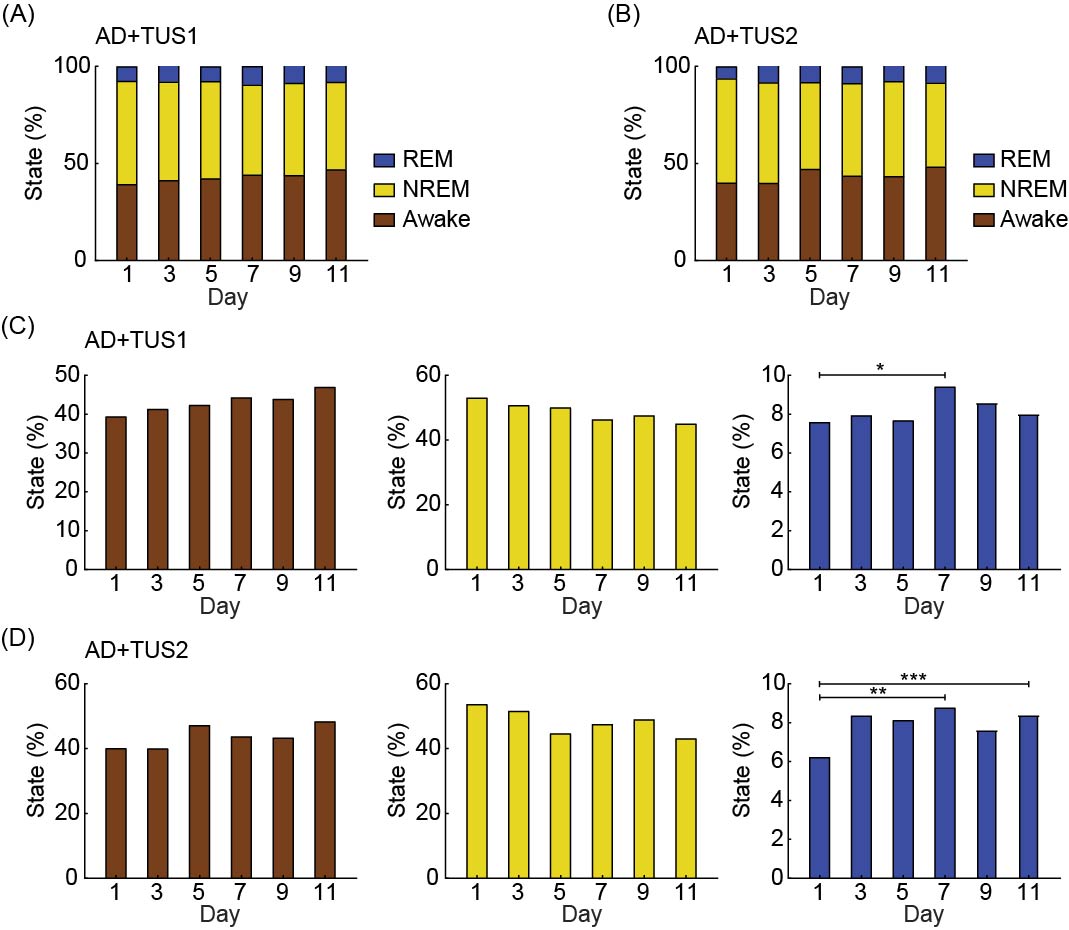
**

**Supplementary Figure 2. Sleep Structure Composition. (A,C)** Percentage of time in Awake, NREM, and REM states in the TUS1 group. **(B,D)** Percentage of time in Awake, NREM, and REM states for the TUS2 group. ANOVA or the Kruskal-Wallis test, followed by Tukey-Kramer post-hoc multiple comparisons. n = 6 mice in each group, *p < 0.05，**p < 0.01.

**
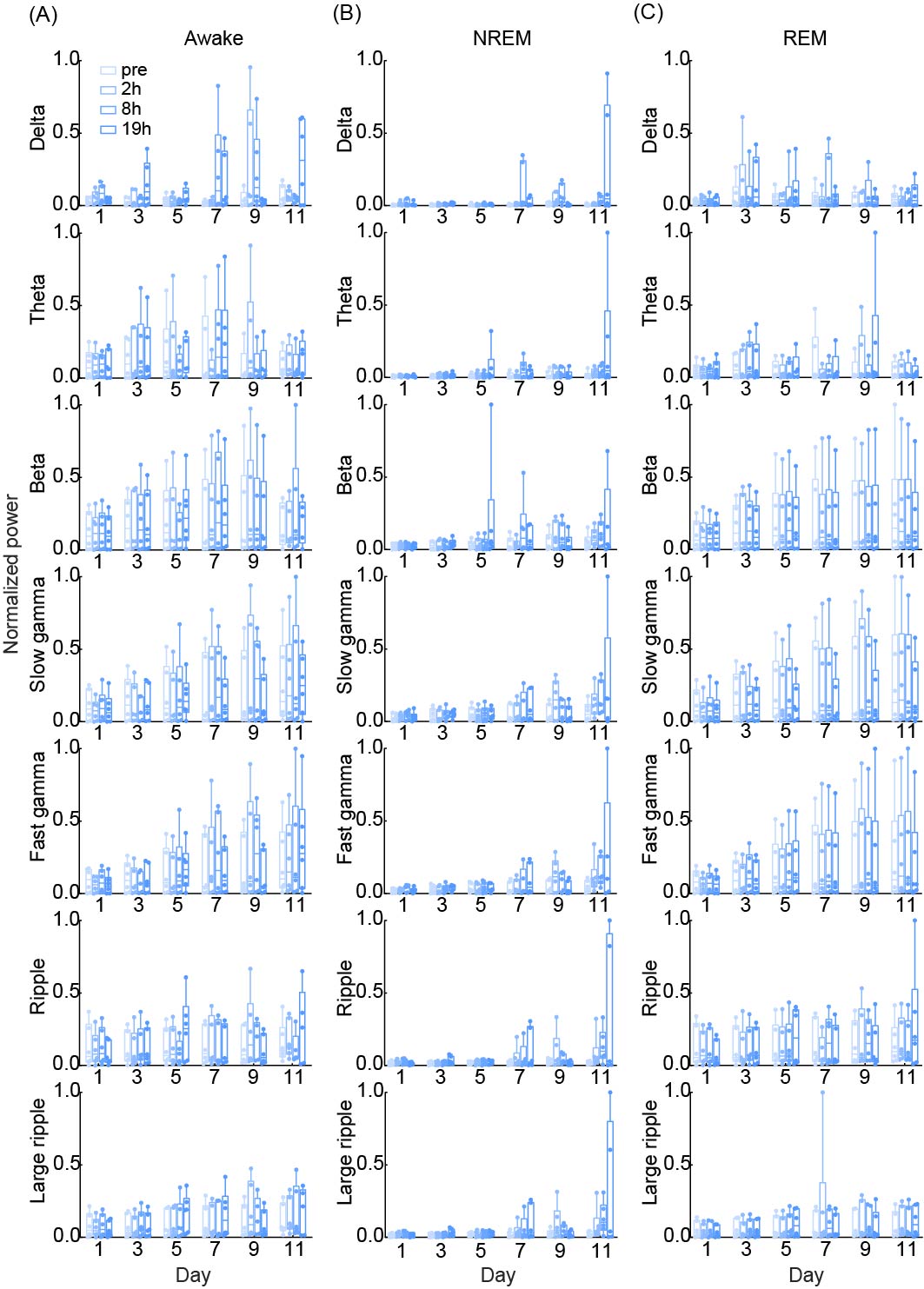
**

**Supplementary Figure 3.** **Data linked to main Figure 3.** Normalized power of different frequency bands in the Awake, NREM, and REM states of the TUS1 group: Delta (0.5-4 Hz), Theta (5-10 Hz), Beta (13-30 Hz), Slow Gamma (30-50 Hz), Fast Gamma (50-100 Hz), Ripple (140-200 Hz), and Large Ripple (100-200 Hz).

**
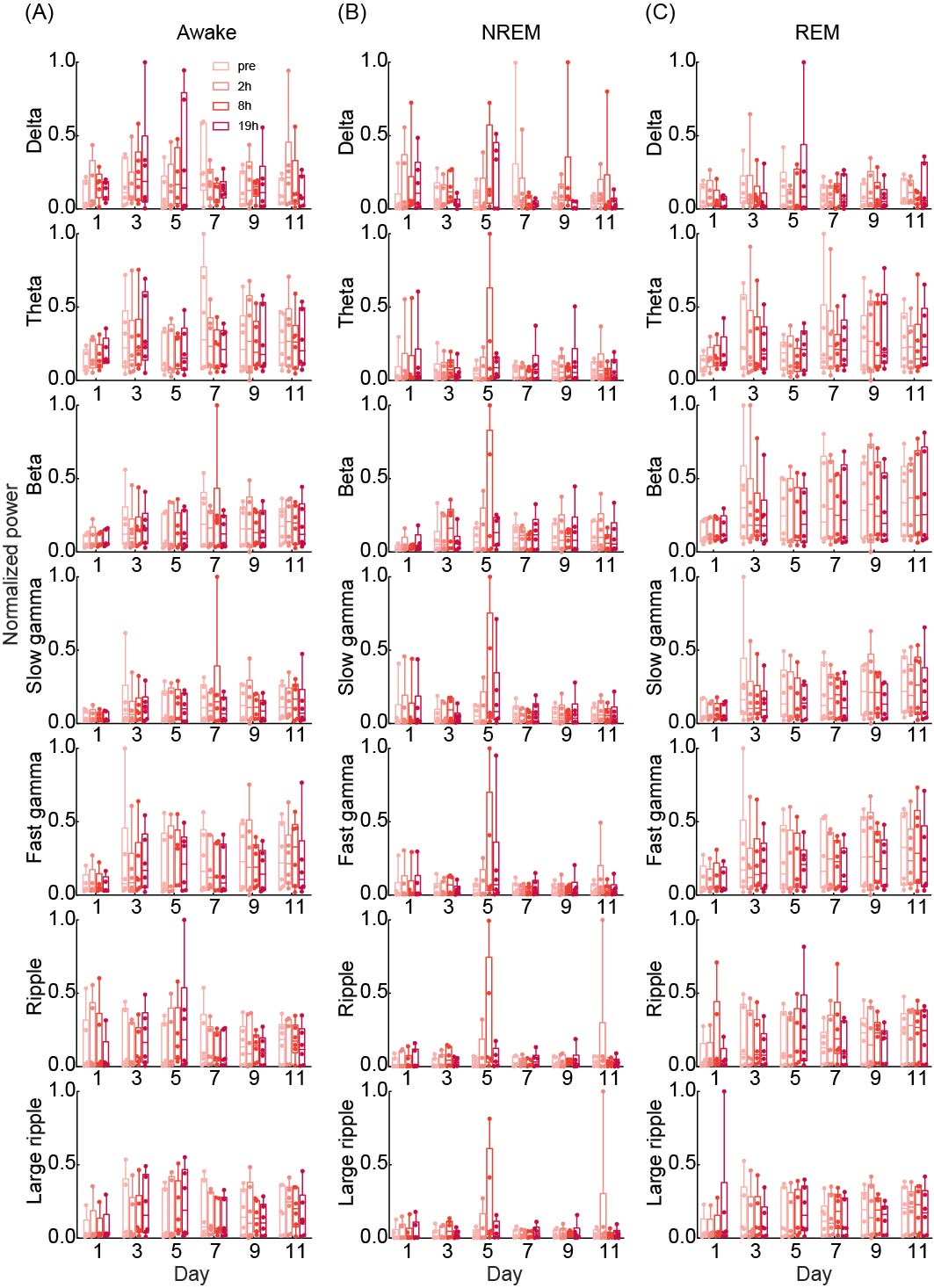
**

**Supplementary Figure 4.** **Data linked to main Figure 3.** Normalized power of different frequency bands in the Awake, NREM, and REM states of the TUS2 group: Delta (0.5-4 Hz), Theta (5-10 Hz), Beta (13-30 Hz), Slow Gamma (30-50 Hz), Fast Gamma (50-100 Hz), Ripple (140-200 Hz), and Large Ripple (100-200 Hz).

**
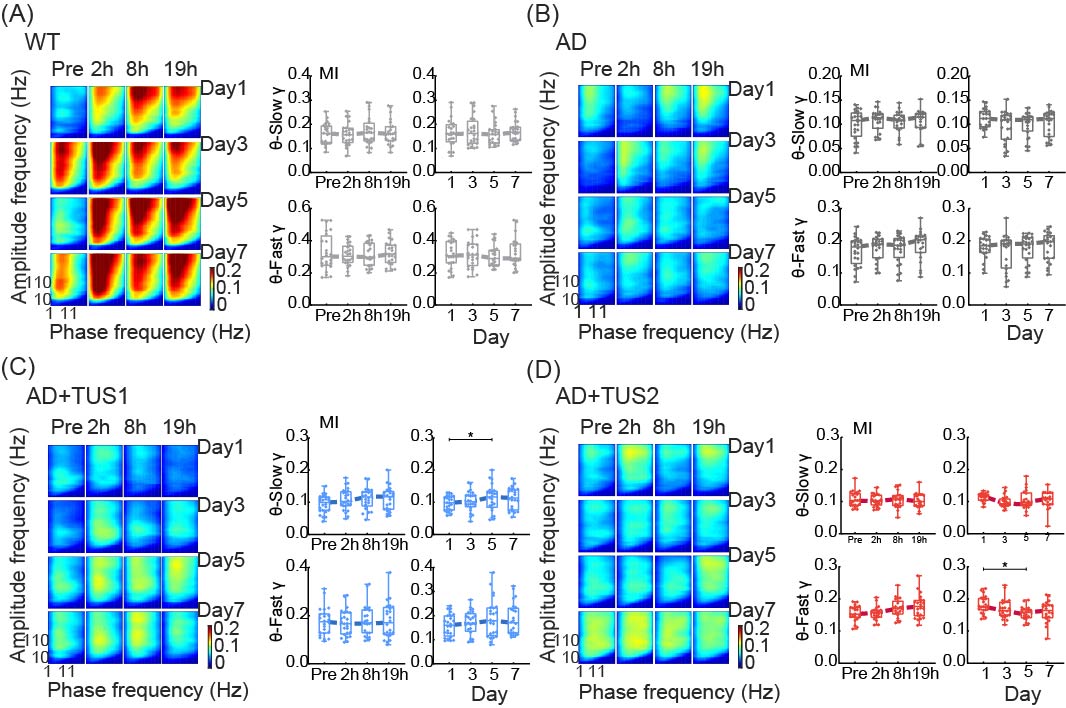
**

**Supplementary Figure 5. Data linked to main Figure 4,** **theta-gamma PAC for all groups without distinguishing states. (A-D)** Left: Images of the modulation index (MI) of phase-amplitude coupling (PAC) for the analytical phase (1-20 Hz) and analytical amplitude (10-200 Hz) across all groups. Right: MI of PAC for theta-slow gamma and theta-fast gamma across different time periods and days. A: Normal group, B: AD group, C: TUS1 group, D: TUS2 group. ANOVA or the Kruskal-Wallis test, followed by Tukey-Kramer post-hoc multiple comparisons. n = 6 mice in each group, *p < 0.05，**p < 0.01.


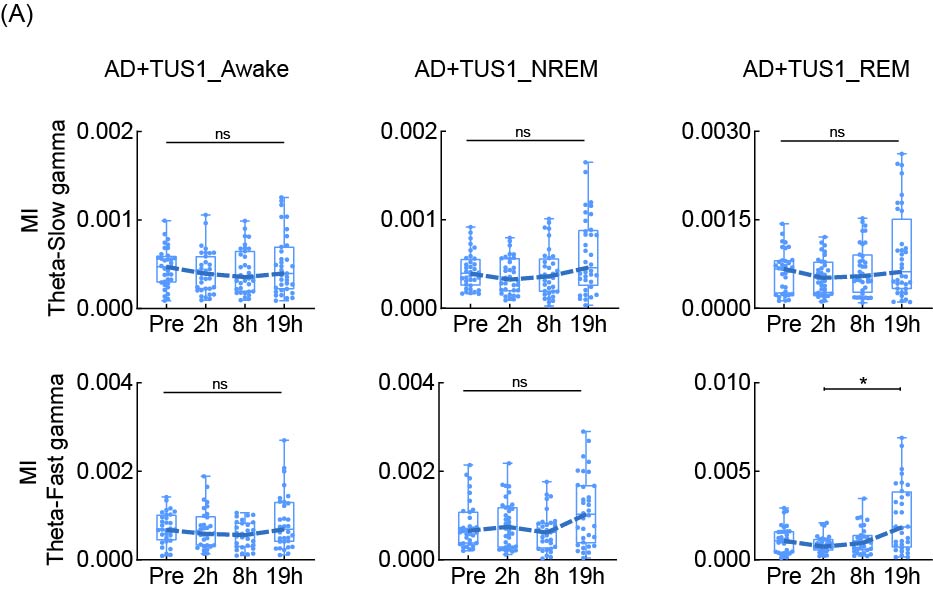


**Supplementary Figure 6. Data linked to main Figure 4,** TUS modulates theta-gamma PAC in CA1. (A) MI of PAC of theta-slow gamma and theta-fast gamma over days in awake, NREM, and REM states. ANOVA or the Kruskal-Wallis test, followed by Tukey-Kramer post-hoc multiple comparisons. n = 6 mice in each group, *p < 0.05.

**
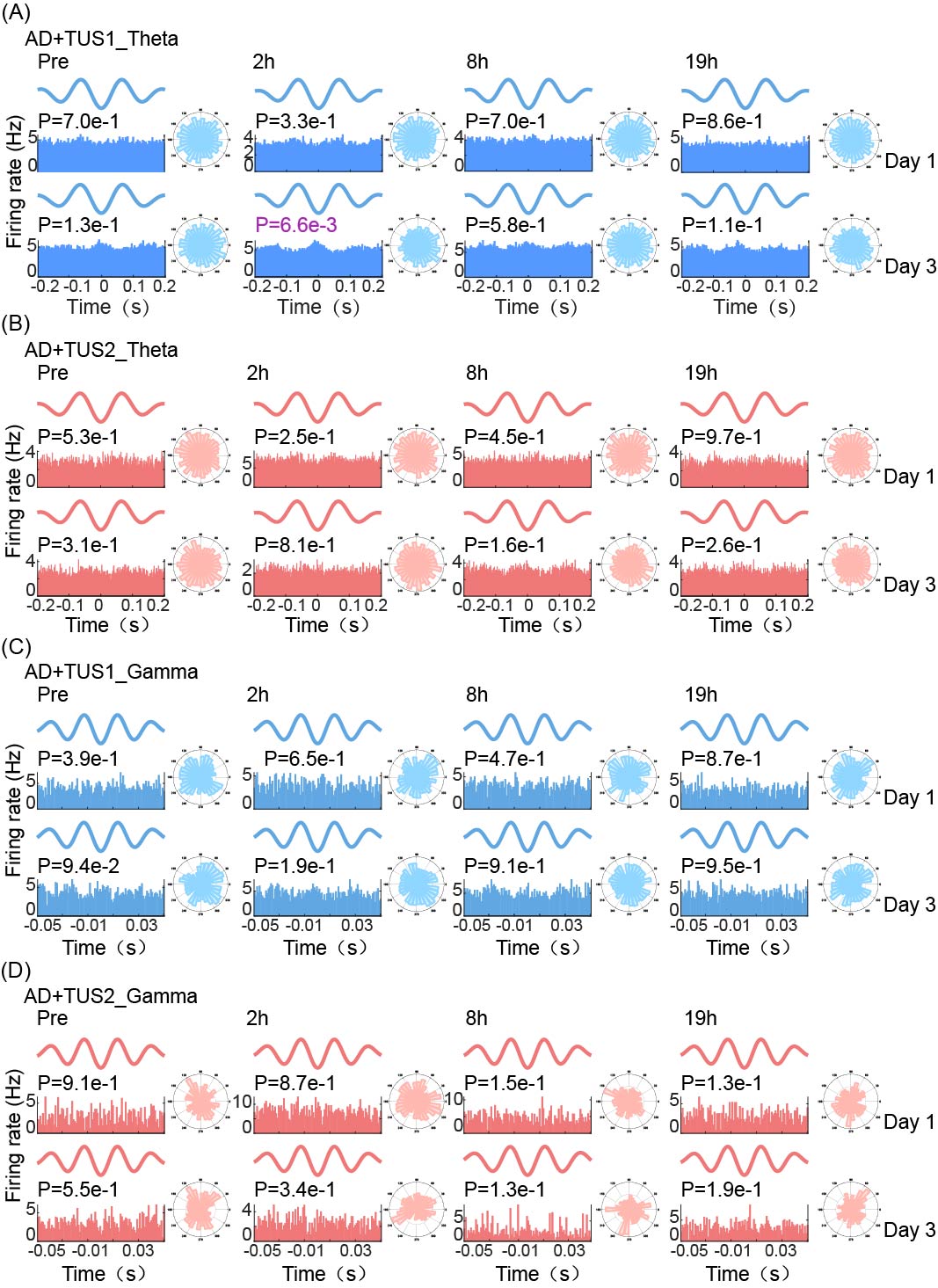
**

**Supplementary Figure 7. Data linked to main Figure 5, phase-locked firing of interneurons to theta/gamma oscillations without distinguishing states on day 1 and day 3 in TUS1 and TUS2 groups.** (A) Phase-locked firing of interneurons to theta oscillations at different time periods on day 1 and day 3 in the TUS1 group (Rayleigh's test, n = 6 mice, *p < 0.05). (B) Phase-locked firing of interneurons to gamma oscillations at different time periods on day 1 and day 3 in the TUS1 group (Rayleigh's test, n = 6 mice, ns: p < 0.05). (C) Phase-locked firing of interneurons to theta oscillations at different time periods on day 1 and day 3 in the TUS2 group (Rayleigh's test, n = 6 mice, ns: p < 0.05). (D) Phase-locked firing of interneurons to gamma oscillations at different time periods on day 1 and day 3 in the TUS2 group (Rayleigh's test, n = 6 mice, ns: p < 0.05).

**
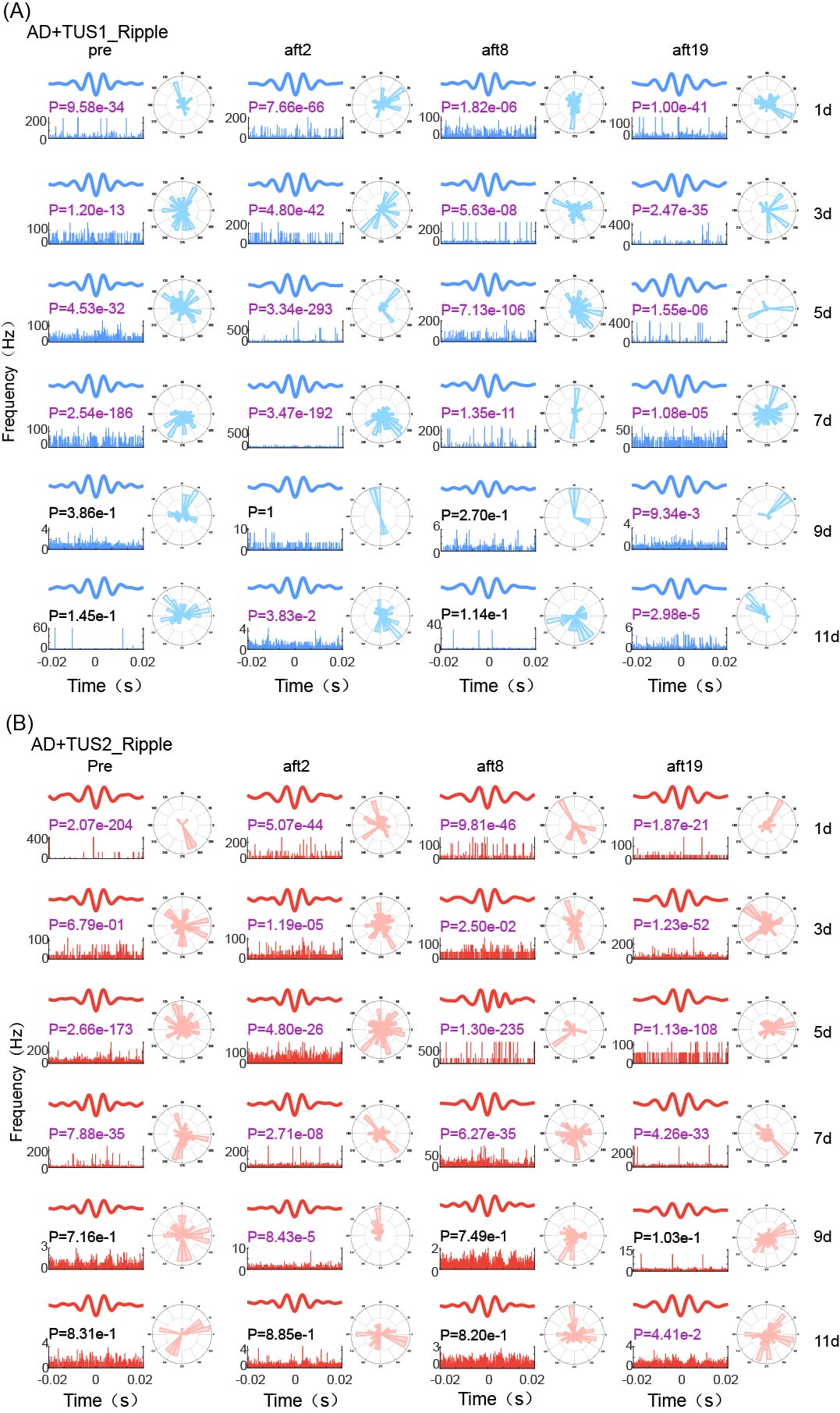
**

**Supplementary Figure 8.** **Data linked to main Figure 5,** phase-locked firing of interneurons to ripple oscillations without distinguishing states following time periods from day 1 to day 11 in TUS1 and TUS2 groups. (A) Phase-locked firing of interneurons to the trough of ripple oscillations in the TUS1 group (Rayleigh's test, n = 6 mice). (B) Phase-locked firing of interneurons to the peak of ripple oscillations in the TUS2 group (Rayleigh's test, n = 6 mice).


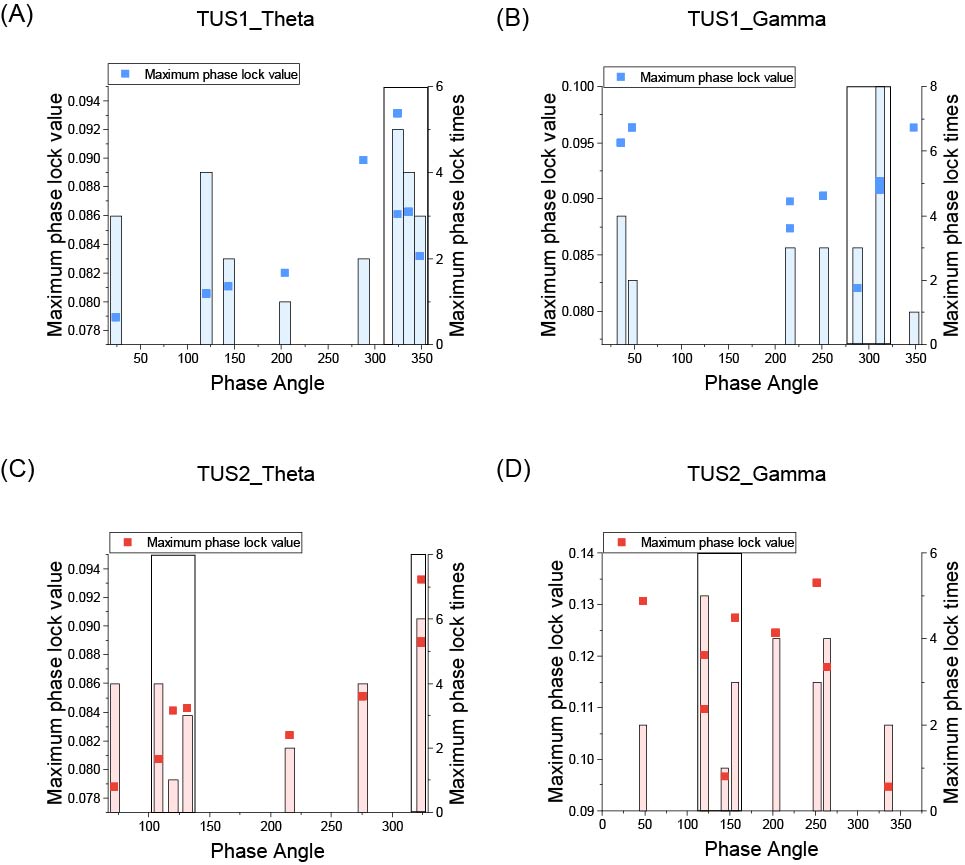


**Supplementary Figure 9. Data linked to main Figure 5, The maximum phase-locking angle and the number of occurrences.** The black box denotes the preferred phase angle of neuron firing in the group.

**
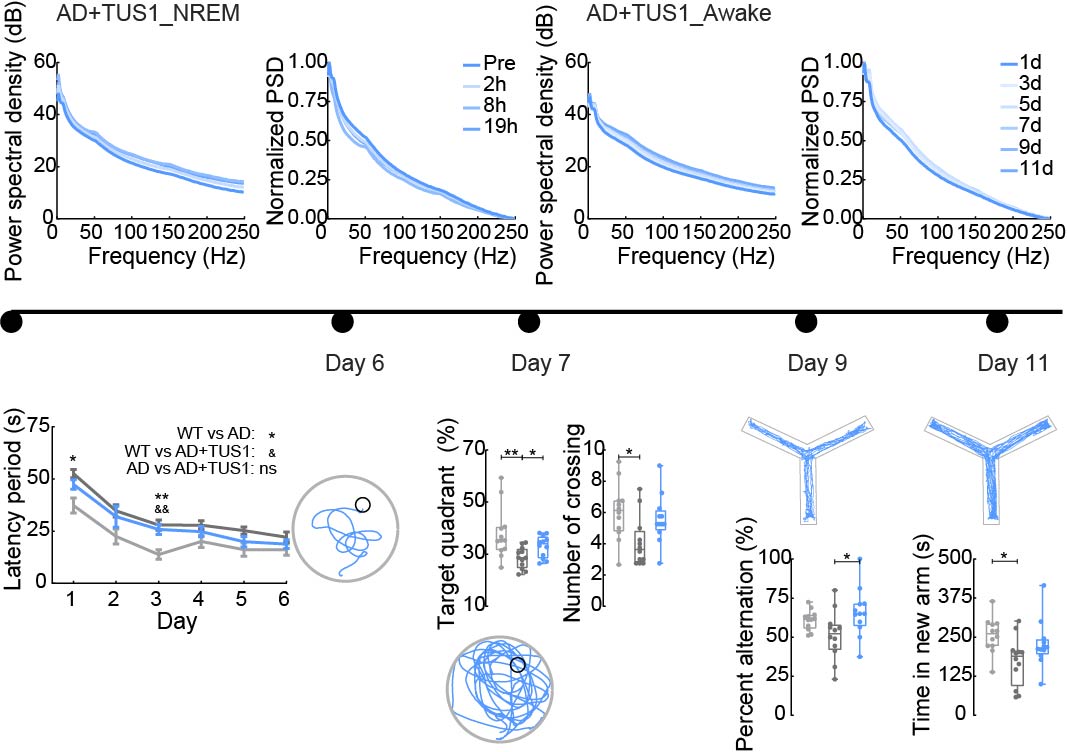
**

**Supplementary Figure 10.** Behavioral results and power spectral density curves to exclude auditory responses on days 1-11. ANOVA or the Kruskal-Wallis test, followed by Tukey-Kramer post-hoc multiple comparisons. n = 6 mice in each group, *p < 0.05，**p < 0.01.

**
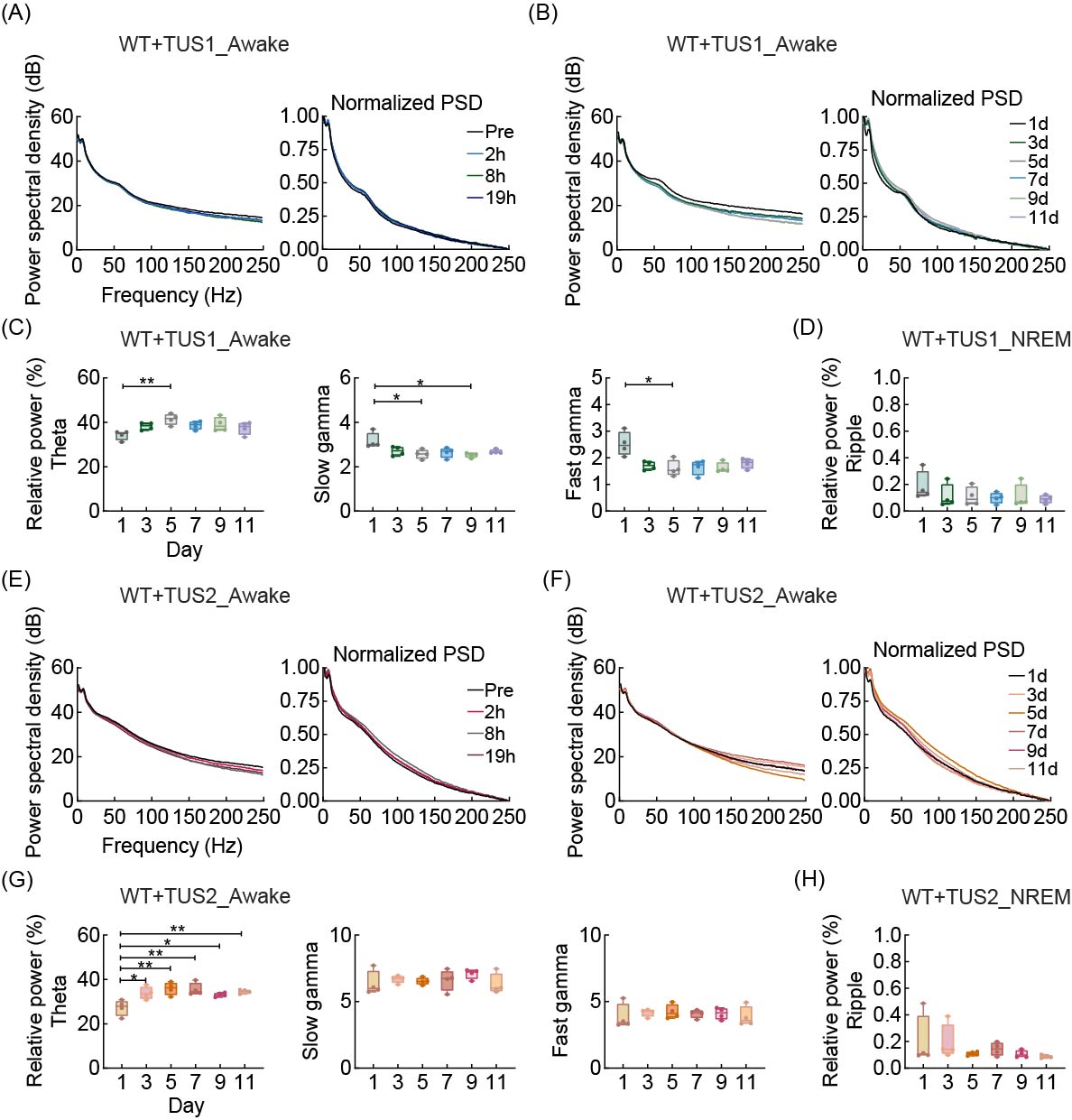
**

**Supplementary Figure 11.** TUS modulates the power of LFP of CA1 neural oscillation in WT mice. (A) Power spectrum curve of LFP in the NREM state in the TUS1 group (Right: normalized PSD curve). (B) Power spectrum curve of LFP in the Awake state in the TUS1 group. (C) Relative power of theta (5-10 Hz), slow gamma (30-50 Hz), and fast gamma (50-100 Hz) in the Awake state of the TUS1 group. (D) Relative power of ripple (140-200 Hz) for NREM states in the TUS1 group. (E) Power spectrum curve of LFP in the NREM state in the TUS2 group (Right: normalized PSD curve). (F) Power spectrum curves of LFP in the NREM state of the TUS2 group. (G) Relative power of theta, slow gamma, and fast gamma oscillations in the Awake state in the TUS2 group. (H) Relative power of ripple oscillation in the NREM states of the TUS2 group. ANOVA or the Kruskal-Wallis test, followed by Tukey-Kramer post-hoc multiple comparisons. n = 4 mice in each group (2 female mice and 2 male mice), *p < 0.05，**p < 0.01.
